# An AMPK activator as a therapeutic option for congenital nephrogenic diabetes insipidus

**DOI:** 10.1172/jci.insight.146419

**Published:** 2021-04-22

**Authors:** Janet D. Klein, Ish Khanna, Ram Pillarisetti, Rachael A. Hagan, Lauren M. LaRocque, Eva L. Rodriguez, Jeff M. Sands

**Affiliations:** 1 Emory University, Department of Medicine, Renal Division, Atlanta, Georgia, USA.; 2 NephroDI Therapeutics, Philadelphia, Pennsylvania, USA.

**Keywords:** Nephrology, Epithelial transport of ions and water

## Abstract

Nephrogenic diabetes insipidus (NDI) patients produce large amounts of dilute urine. NDI can be congenital, resulting from mutations in the type-2 vasopressin receptor (V2R), or acquired, resulting from medications such as lithium. There are no effective treatment options for NDI. Activation of PKA is disrupted in both congenital and acquired NDI, resulting in decreased aquaporin-2 phosphorylation and water reabsorption. We show that adenosine monophosphate–activated protein kinase (AMPK) also phosphorylates aquaporin-2. We identified an activator of AMPK, NDI-5033, and we tested its ability to increase urine concentration in animal models of NDI. NDI-5033 increased AMPK phosphorylation by 2.5-fold, confirming activation. It increased urine osmolality in tolvaptan-treated NDI rats by 30%–50% and in V2R-KO mice by 50%. Metformin, another AMPK activator, can cause hypoglycemia, which makes it a risky option for treating NDI patients, especially children. Rats with NDI receiving NDI-5033 showed no hypoglycemia in a calorie-restricted, exercise protocol. Congenital NDI therapy needs to be effective long-term. We administered NDI-5033 for 3 weeks and saw no reduction in efficacy. We conclude that NDI-5033 can improve urine concentration in animals with NDI and holds promise as a potential therapy for patients with congenital NDI due to V2R mutations.

## Introduction

The production of concentrated urine occurs in the renal medulla (1). Vasopressin is the key hormonal regulator and stimulates urine concentration by (a) increasing medullary interstitial osmolality by increasing NaCl reabsorption through the Na-K-2Cl cotransporter-2 (NKCC2) in the thick ascending limb of the loop of Henle and (b) increasing water reabsorption by increasing aquaporin-2 (AQP2) water channel accumulation in the collecting duct. Vasopressin further increases inner medullary interstitial osmolality when it increases urea transport by phosphorylating the urea transporter-A1 (UT-A1) in the inner medullary collecting duct (IMCD). When there is a need to conserve water, vasopressin is secreted by the posterior pituitary and stimulates osmotic water reabsorption along the entire collecting duct, driven by the hypertonic medullary interstitium.

Diabetes insipidus (DI) is a disease in which a patient produces very large quantities of dilute urine (2, 3). It can result from failure of the posterior pituitary to make or secrete vasopressin (central DI) or from a failure of the kidney to respond to vasopressin (nephrogenic DI; NDI). NDI can be congenital, due to mutations in the type 2 vasopressin receptor (V2R) or AQP2, or it can be acquired, most commonly from lithium therapy. In total, 90% of patients with congenital NDI have a mutation in their V2R, which is located on the X-chromosome. Patients with congenital NDI urinate up to 10–20 liters in a day and must drink an equal quantity of fluid to avoid dehydration. Failure to do so can cause mental retardation due to repeated episodes of severe dehydration. Some children require a feeding tube in order to provide them with enough water intake to match their urine output. Although the disease is currently better recognized early in life so that episodes of dehydration can be avoided, the necessity for frequent and high-volume urination can lead to bladder dysfunction, reflux nephropathy, and chronic kidney disease. Conventional treatment options, such as indomethacin, thiazides, and salt restriction, are only minimally effective.

We previously showed that Metformin improves urine concentrating ability in 2 rodent models of X-linked NDI: rats treated with tolvaptan (a V2R inhibitor) and inducible V2R-KO mice (4, 5). Metformin is an activator of AMPK, but also has AMPK-independent mechanisms, such as inhibition of mitochondrial respiration, inhibition of mitochondrial glycerophosphate dehydrogenase, and a mechanism involving the lysosome (6). In addition, Metformin is a weak and nonselective AMPK activator, and the amount of Metformin used in our previous animal studies is well above the equivalent maximal allowable dose for human use. The data from our Metformin study, however, inspired us to seek a potent and orally active AMPK activator as a potential therapy for NDI due to V2R mutations. Since Metformin is an oral hypoglycemic agent that is used to treat type 2 diabetes mellitus, we also sought an AMPK activator that did not cause hypoglycemia. We carried out extensive medicinal chemistry work, developed a potentially novel series of potent and selective AMPK activators, and found that NDI-5033 did not reduce blood sugar in normal animals (7). The purpose of the present study is to evaluate whether NDI-5033 could improve urine concentrating ability in 2 rodent models of NDI.

## Results

### Medicinal chemistry efforts and identification of NDI-5033 as a lead candidate

Based on our earlier work with Metformin (4, 5), we sought more potent and selective AMPK activators to confirm efficacy in multiple models of X-linked congenital NDI. During exploratory efforts to identify a potentially novel series of AMPK activators, we came across reports of fatty acid–derived molecules with hints of efficacy in preclinical models of dyslipidemia and insulin sensitization. These compounds were weak activators of AMPK (EC_50_, 75–250 μM) with nonoptimal oral pharmacokinetics (PK) profiles (8–11). They were also structurally distinct from the known AMPK activators. Applying principles of medicinal chemistry, we synthesized select compounds to incorporate the pharmacophore, which resulted in potent AMPK activators (EC_50_, 1–3 μM). Encouraged by the data, we carried out extensive structure-activity relationship studies and physicochemical property optimization to help boost potency, oral absorption, and tissue distribution of the molecules. These efforts culminated in the generation of a library of potent, selective, diverse AMPK activators (7) and identification of NDI-5033 as a lead candidate for potential treatment of NDI. NDI-5033 is a potentially novel, achiral organic molecule with molecular descriptors (H-bond donors = 4, H-bond acceptors = 4, molecular weight ≤ 400, calculated partition coefficient [cLogP] = 4.6, polar surface area [PSA] = 86.2) that favor good oral absorption.

### Activation of AMPK by NDI-5033

**Figure 1:**
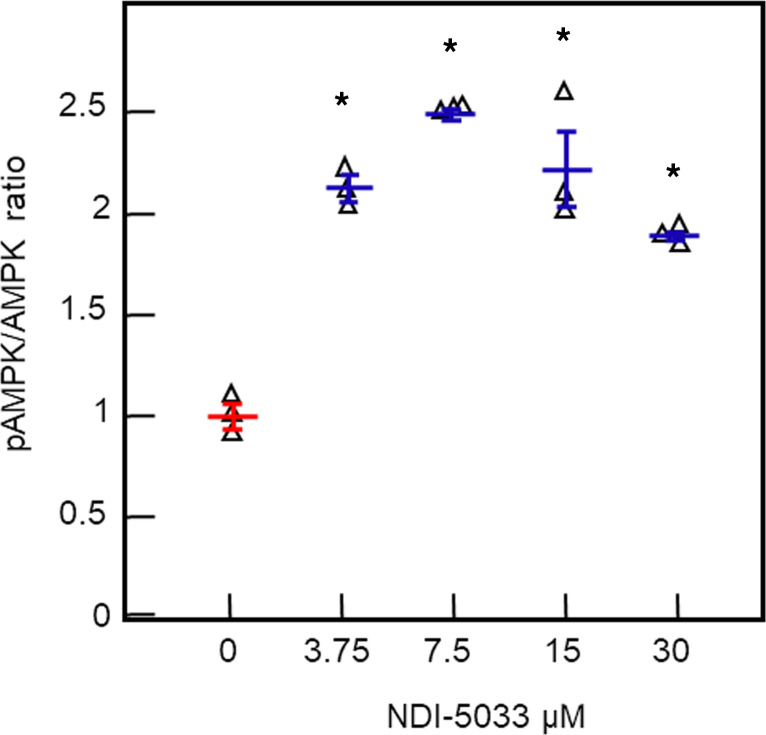
NDI-5033 stimulates AMPK phosphorylation in HEK-293–cultured kidney cells. HEK-293 cells were cultured to confluence and then incubated with NDI-5033 at doses of 0 (vehicle control), 3.75, 7.5, 15, and 30 μM for 6 hours. Cells were lysed and assayed for pThr172AMPK and total AMPK by ELISA. Mean ± SEM, *n =* 3. *P < 0.001 versus vehicle control by ANOVA followed by a Tukey-Kramer multiple-comparisons test.

To establish that NDI-5033 activates AMPK, we treated HEK-293 kidney cells with doses of NDI-5033 from 0 μM (vehicle) to 30 μM and measured the pThr172-AMPK and total AMPK levels by ELISA (Figure 1). There was a statistically significant increase in the phosphorylation of AMPK at 3.75 μM NDI-5033 with 2.5-fold increase using 7.5 μM drug. There was a 1.9-fold increase in the phosphorylation of AMPK over the vehicle control in response to 30 μM NDI-5033. This trend was observed in 2 replicate experiments.

### Efficacy studies

#### Determination of the optimal dose for NDI-5033(P1).

**Figure 2:**
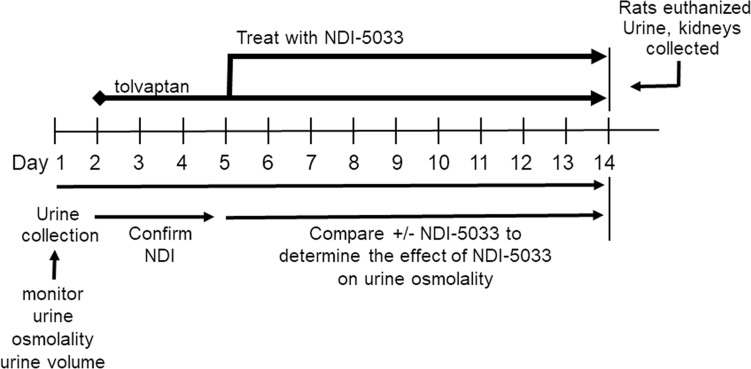
Protocol plan for treatment of rats with NDI-5033. Urine was collected for a period of 24 hours before initiation of tolvaptan treatment. Tolvaptan (10 mg/kg/day) was delivered by gavage feeding daily. Osmolality was monitored for 3 days to verify development of NDI. NDI-5033 treatment by gavage feeding was initiated and given daily for up to 14 total study days, with daily urine collection for osmolality determination.

**Figure 3:**
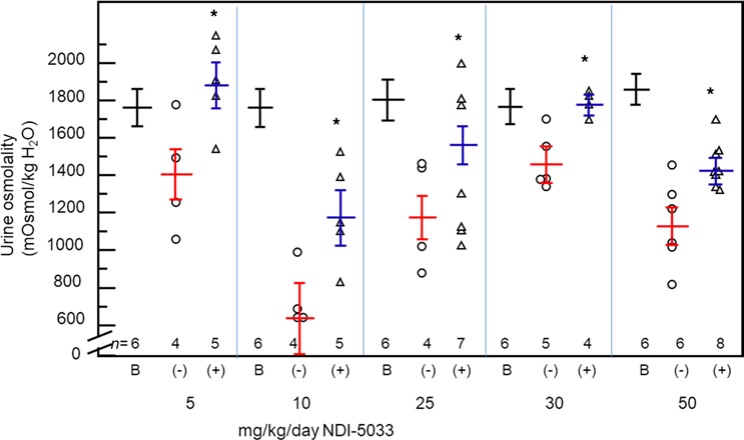
Urine osmolality increases with NDI-5033(P1) treatment. Tolvaptan (10 mg/kg/day) was delivered by gavage feeding daily. Osmolality was monitored for 3 days to verify development of NDI; then, NDI-5033 was administered at doses of 5, 10, 25, 30, and 50 mg/kg/day by gavage feeding daily for an additional 7 days with daily urine collection for osmolality determination. B, basal untreated; (–), control rats receiving tolvaptan only; (+), osmolality of rats treated with both tolvaptan and NDI-5033 at the indicated doses. Urine osmolalities from the final 24-hour urine collection. Mean ± SEM. *P < 0.05 by unpaired Student’s *t* test comparing tolvaptan-treated animals receiving NDI-5033 (+) with those not receiving NDI-5033 (–).

The experimental protocol for determining the dose response for NDI-5033 is outlined in Figure 2. Urine was collected for 24 hours before initiation of tolvaptan treatment. Tolvaptan (10 mg/kg/day) was delivered by gavage feeding to rats daily. Development of NDI by tolvaptan treatment occurred in 3 or 4 days. Since the normal size of a group was 5–8 rats, a single experiment usually compared control and a single dose of NDI-5033. As a consequence, each dose of NDI-5033 is compared with the control rats that were part of that same experiment. Osmolality was monitored for 3 days to verify development of NDI; then NDI-5033 was administered by gavage feeding daily for an additional 7 days. Figure 3 provides the average maximal urine osmolalities for drug-treated NDI rats at doses of 5, 10, 25, 30, and 50 mg/kg/day and the control rats from the same day. Basal urine osmolalities are shown, as well. All doses produced a significant improvement in urine concentration in the NDI rats, despite a considerable degree of animal-to-animal variability on any given study day.

#### Efficacy in the absence of a V2R receptor.

**Figure 4:**
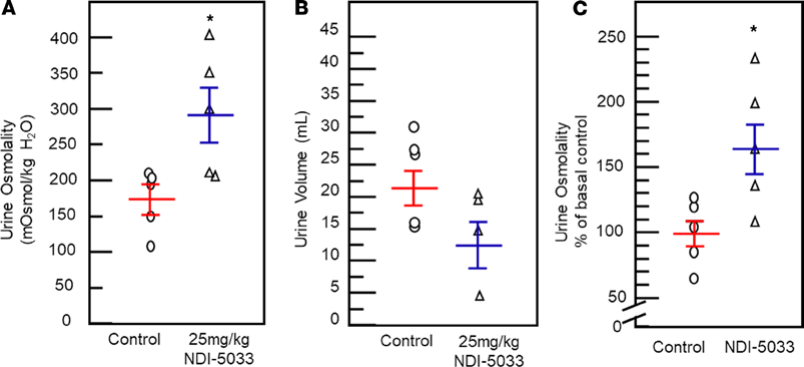
V2R-KO mice show improved urine concentration with NDI-5033(P1). (**A** and **B**) Urine osmolality (24-hour urine collection from day 2) (**A**) and urine volume (**B**) for control V2R-KO mice before treatment and after receiving NDI-5033 (25 mg/kg/day) for 2 days. Mean ± SEM, *n =* 5. (**C**) The experiment was replicated, and the combined data from 2 trials are shown as the percent of control. Mean ± SEM, *n =* 4–5. *P < 0.05 by unpaired Student’s *t* test.

Because our NDI model with tolvaptan relies on its binding to and blocking the V2R, we considered whether NDI-5033 could be displacing the tolvaptan, thus removing the blockage and restoring urine osmolality as a consequence. To investigate this, we examined the effect of NDI-5033(P1) on an inducible V2R-KO mouse (4). Removal of the V2R is initiated by tamoxifen feeding. When urine osmolality dropped to 300 mOsM or below, the mouse was given NDI-5033(P1) at 25 mg/kg/day by i.p. injection (Figure 4A). Urine was collected over 24 hours for 2 days, after which the animal was euthanized. There was a significant increase in urine osmolality from 177 ± 19 mOsM (control) to 290 ± 34 mOsM (NDI-5033–treated). The experiment was repeated at 50 mg/kg/day with similar results. Urine volume decreased, although the change was not statistically significant (Figure 4B). Figure 4C shows the 50% increase in urine osmolality with NDI-5033 in the absence of a functional V2R receptor.

#### AMPK activation and hypoglycemia.

**Figure 5:**
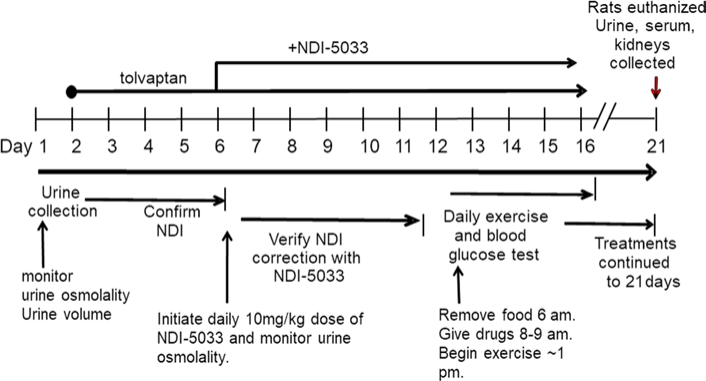
Protocol plan for determining effect of NDI-5033 on blood glucose levels. Urine was collected over 24 hours; then, tolvaptan (10 mg/kg/day) was delivered by gavage feeding daily. Osmolality was monitored for 3 days to verify development of NDI. NDI-5033 treatment by gavage feeding was initiated and given daily for up to 20 days, with daily urine collection for osmolality determination. On day 12, food was removed, mice ere exercised for 30 minutes, NDI-5033 was given, and then food was restored. This was repeated for 5 exercise days. Blood glucose was determined immediately before and after exercise.

**Figure 6:**
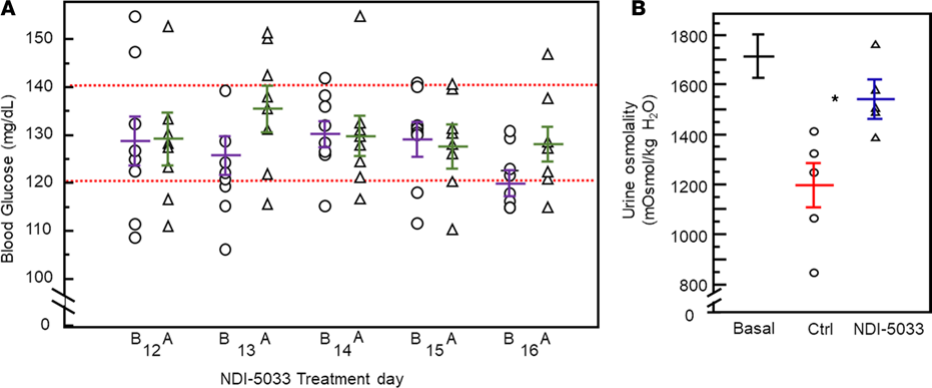
Exercised rats receiving NDI-5033 do not experience hypoglycemia. (**A**) Blood Glucose levels. B, before exercise; A, after exercise. All values shown are from rats receiving NDI-5033; red dashed lines show normal range of blood glucose for rats. Mean ± SEM. (**B**) Urine osmolalities of rats before treatment (basal, *n =* 16), as well as control (Ctrl, tolvaptan-only treatment) and drug-treated (Tolvaptan + NDI-5033) rats averaged over the 5 days when exercised. Mean ± SEM, *n =* 5. *P < 0.05 by unpaired Student’s *t* test comparing tolvaptan-treated animals receiving NDI-5033 (+) with those not receiving NDI-5033 (–).

A potential side effect of treating patients with AMPK activators such as Metformin is hypoglycemia, especially in the setting of caloric restriction. We investigated whether NDI-5033 showed evidence of producing hypoglycemia. We designed an experiment to mimic conditions where hypoglycemia was most likely to occur: caloric restriction with exercise (Figure 5). Rats were allowed to eat overnight; then, food was removed. They were treated with NDI-5033(P1), and 3 hours later, they were exercised by treadmill running for 30 minutes. Blood glucose was sampled immediately before and after the bout of exercise. As shown in Figure 6A, exercising rats that were treated with NDI-5033 did not influence blood glucose levels. Urine osmolalities of the NDI rats with and without NDI-5033 confirmed that the drug was effective on exercise days (Figure 6B); basal urine osmolalities are shown, as well.

**Figure 7:**
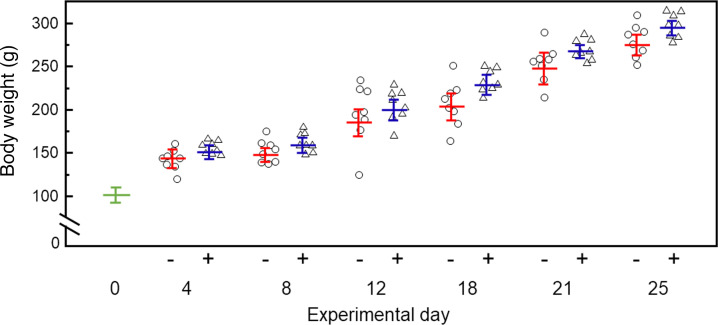
Rat body weight with time. Day 0 indicates weight before any tolvaptan (*n =* 16). All rats were treated with tolvaptan beginning day 0, and half were treated with tolvaptan and 10 mg/kg NDI-5033 beginning day 4. Mean ± SEM, *n =* 8 per group; – indicates rats receiving only tolvaptan; + indicates rats receiving tolvaptan and NDI-5033.

To ensure that the extended treatment time and the disrupted feeding regimen did not adversely affect the rat’s general health, we followed body weight over the duration of the experiment (Figure 7). Body weight was not different between tolvaptan-induced NDI rats and rats receiving both tolvaptan and NDI-5033. A slight increase in body weight in the drug-treated rats relative to those without drug developed over time, but this did not reach statistical significance.

### Studies using NDI-5033 from manufacturing process 2

#### PK profile on oral dosing.

NDI-5033 was prepared using a revised manufacturing process (process 2, P2) to improve scale-up of the molecule and translational capacity for future human studies. NDI-5033(P2) shows very high bioavailability (>85%) on oral dosing (3–10 mg/kg) in Sprague Dawley rats. I.v. dosing of NDI-5033 gave an elimination half-life of 6.9 hours, a low clearance of 2.16 L/h/kg, and a volume of distribution of 8.39 L/kg (Table 1). The plasma levels (AUC, C_max_) display a dose-dependent increase from 3 to 10 mg/kg oral dosing.

NDI-5033(P2) was used to study oral bioavailability (Table 1) and tissue distribution (Table 2) on single oral dosing. NDI-5033(P2) displayed superior oral bioavailability compared with NDI-5033(P1), possibly linked to improved dissolution of NDI-5033(P2) in the lipid formulation.

#### Tissue distribution of NDI-5033(P2).

After a single oral dose (15 mg/kg) in Sprague Dawley rats, the plasma and tissue levels (liver, kidney) of NDI-5033 were assessed over 24-hour time points (0.5, 1, 2, 4, 6, and 24 hours). NDI-5033 absorbs and distributes to kidney rapidly (time of maximum plasma concentration [T_max_] = 1 hours) and shows very good levels (AUC_last_ = 9.57 μg × h/mL) in the kidney (Table 2). The kinetics of elimination of NDI-5033 from the plasma and the kidney compartments follow a similar pattern.

#### Determination of the optimal dose for NDI-5033(P2).

**Figure 8:**
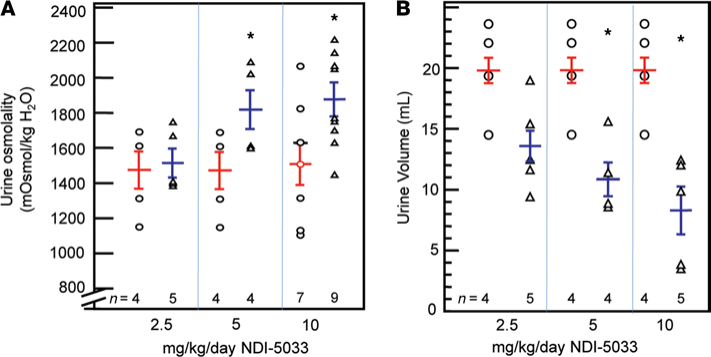
Urine osmolality increases and urine volume decreases with NDI-5033(P2) treatment. Tolvaptan (10 mg/kg/day) was delivered by gavage feeding daily. (**A**) Combined data from 2 experiments. Osmolality was monitored for 3 days to verify development of NDI; then, NDI-5033 treatment at doses of 2.5, 5, and 10 mg/kg/day was initiated and given daily for 5 additional days, with daily urine collection for osmolality determination. Red data (left in each pair) show control levels in rats receiving tolvaptan only. Blue data (right data) show urine osmolality from tolvaptan-treated rats receiving NDI-5033 at the doses indicated. Mean ± SEM. *P < 0.05 versus the untreated control values by unpaired Student’s *t* test. (**B**) Urine volume from one of the experiments from **A**. Red data (left in each pair) show control levels in rats receiving tolvaptan only. Blue data (right data) show 24-hour urine volumes from tolvaptan-treated rats receiving NDI-5033 at the doses indicated. Mean ± SEM. *P < 0.05 versus 0 (vehicle control) by ANOVA followed by a Tukey-Kramer multiple-comparisons test.

NDI-5033(P2) required a new vehicle for solubilization. Using oral NDI-5033(P2) in a lipid mixture containing 20% transcutol, 40% labrasol, and 40% labrafil, we repeated a dose-response determination using NDI-5033(P2) doses of 2.5, 5, and 10 mg/kg/day, again in the tolvaptan-treated rat NDI model, as described in Figure 2. The control group received the lipid vehicle. NDI-5033(P2) effectively improved urine osmolality (Figure 8A) and reduced urine volume (Figure 8B) at both 5 and 10 mg/kg/day.

#### Long-term effectiveness of NDI-5033.

**Figure 9:**
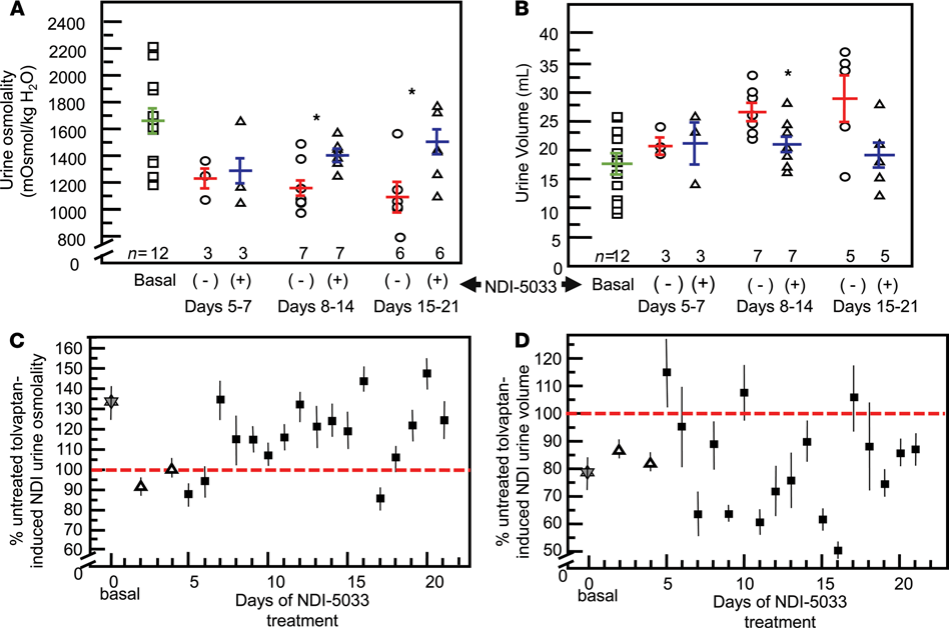
Efficacy of NDI-5033 persists for 21 days. Rats were treated with tolvaptan (10 mg/kg/day) to produce NDI; then, half of the animals were designated as the treatment group and received NDI-5033 at 10 mg/kg/day beginning on day 5 until day 21. Urine osmolalities were determined daily. (**A** and **B**) Data show weekly average urine osmolalities (**A**) and 24-hour urine volumes (**B**) determined from daily average osmolalities (6–7 rats per group) for tolvaptan treated (red) and tolvaptan-treated rats receiving NDI-5033 (blue). Mean ± SEM. *P < 0.05 by unpaired Student’s *t* test comparing tolvaptan-treated animals receiving NDI-5033 (+) with those not receiving NDI-5033 (–). (**C**) Daily average urine osmolalities as a percentage of the average tolvaptan-treated controls designated at 100% (red dashed line). Gray star, basal osmolality before tolvaptan initiation; open triangles, tolvaptan-only initiation of NDI; solid squares, NDI rats treated with NDI-5033. Mean ± SEM. (**D**) Daily average 24-hour urine volumes as a percentage of the average tolvaptan-treated controls designated at 100% (red dashed line). Gray star, basal 24-hour urine volume before tolvaptan initiation; open triangles, tolvaptan-only initiation of NDI; solid squares, NDI rats treated with NDI-5033. Mean ± SEM.

Rats were treated with tolvaptan (10 mg/kg/day) to produce NDI; then, half of the animals received NDI-5033 at 10 mg/kg/day beginning on day 5 until day 21. Urine osmolalities were determined daily. Weekly average urine osmolalities, determined from daily average osmolalities for tolvaptan-treated rats and tolvaptan-treated rats receiving NDI-5033, revealed significant improvement in the urine osmolalities over both weeks 2 and 3 in this series (Figure 9A). Urine volume decreased in weeks 2 and 3, although the decrease was statistically significant in week 2 but not week 3 (Figure 9B). The urine osmolality and volume results are shown in a different form in Figure 9, C and D, respectively, which provide the daily average urine osmolalities and volumes as a percentage of the average tolvaptan-treated controls designated at 100%. In this form, it is clear that most of the 21 treatment days showed an improvement in urine concentration with NDI-5033 when compared with the contemporaneous untreated animals.

## Discussion

We previously showed that Metformin improved urine concentration in 2 rodent models of X-linked congenital NDI and proposed that this beneficial effect was mediated by AMPK (4, 5). The data reported in this study show that NDI-5033, a potent and selective AMPK activator (7), improves urine concentration in both our tolvaptan-treated rat model of NDI and in inducible V2R-KO mice. The beneficial effect of NDI-5033 was sustained for up to 21 days. Importantly, NDI-5033 did not change blood sugar, either before or after exercise. These results suggest that NDI-5033 may be a novel treatment option for X-linked congenital NDI and potentially for other types of NDI, as well.

Congenital NDI is a life-long condition, and the polyuria does not diminish over time (3, 12, 13). Thus, any treatment needs to be effective long-term. We tested the efficacy of NDI-5033 for 3 weeks. We did not see any diminution in benefit in tolvaptan-treated rats receiving NDI-5033 for 3 weeks, and the rats seemed to tolerate NDI-5033 without any obvious negative effects, as the increase in body weight over treatment time attests. The rats that received NDI-5033 had a slightly larger weight, suggesting that these animals may have been less dehydrated, but the weight difference was not statistically significant. Thus, the beneficial effect of NDI-5033 is sustained over the entire 3-week study. The rats were gavage fed once daily in the present studies. If the half-life of the drug is less than 24 hours, we may be underestimating the beneficial effect of NDI-5033 since the urine was collected over 24 hours.

X-linked congenital NDI is caused by mutations in the V2R in 90% of patients (2, 3). It is a rare disease with an estimated prevalence of 8.8 per million baby boys born (13). Baby boys can present as early as 1 week of age with severe dehydration, hypernatremia, and hyperthermia (13). If the condition is diagnosed promptly and the boys are treated with abundant water intake, they have a normal lifespan and physical and cognitive development (13). However, if this does not occur, they can develop mental retardation due to repeated episodes of severe dehydration (13). Conventional therapy is to match water intake to urine output, along with a very low–sodium diet (0.5 g/day). Patients are often prescribed a thiazide diuretic, with or without a prostaglandin synthetase inhibitor, or amiloride (12). Indomethacin and tolmetin seem to be more effective than ibuprofen (12), although none of the pharmacological therapies are particularly effective and all are potentially nephrotoxic.

Other medications that have been studied as therapies for NDI in rodent models — such as sildenafil (14, 15), erlotinib (16), statins (17–19), ONO-AE1-329 (an EP4 prostanoid receptor agonist; ref. 20), and clopidogrel (21) — have shown some short-term benefit (hours). However, the beneficial effects are generally gone within a day, and some of these agents have side effects that would preclude life-long therapy. Acetazolamide was studied for lithium-induced NDI in rodents, but it did not improve urine concentrating ability and had side effects when used for 1–2 weeks (22, 23). Since congenital NDI is a life-long condition and these potential therapies do not show a sustained effect, they are less likely to be useful clinically than NDI-5033.

An important consideration for a potential therapy for NDI is whether NDI-5033 caused hypoglycemia. Metformin is an oral hypoglycemic medication that is used to treat type 2 diabetes mellitus. If NDI-5033 caused hypoglycemia, it would not be optimal as a therapy for congenital NDI, as it would be risky to treat patients, especially children, with a mediation that lowers blood sugar. Children attend school and then often play after school. They may not eat while playing, and their play may involve exercise. To mimic this condition, we removed food from the rats in the morning and then exercised them for 30 minutes in the afternoon. Their blood sugar remained in the normal range both before and after exercise. Thus, we concluded that NDI-5033 is unlikely to cause hypoglycemia, even in fasted, exercising children.

In summary, we show that AMPK activation by NDI-5033 increases urine-concentrating ability in tolvaptan-treated rats and V2R-KO mice. The effect persists for at least 3 weeks, and there is no evidence of hypoglycemia, even when the rats are fasted and exercised. NDI-5033 may become a therapy for patients with congenital NDI due to V2R mutations.

## Methods

### AMPK activity assay (pAMPK/AMPK ratio) in HEK cells.

For cell culture experiments, a 10 mM stock solution of NDI-5033 (NephroDI Therapeutics) was prepared in DMSO and diluted into culture medium. In the present study, NDI-5033 was tested at various concentrations for its ability to activate AMPK in a kidney cell line, HEK-293 cells (American Type Culture Collection; ATCC). The assay was carried out as per the instructions provided in the kit manual (CisBioassays, catalog 64MPKPEG). Total and pAMPK levels were detected in cell lysates using the kit reagents. The total AMPK is detected in a sandwich assay format using 2 different specific antibodies, 1 labeled with Eu3+-cryptate (Eu3+-cryptate; donor) and the second with d2 (acceptor). On day 1, HEK-293 cells were seeded (5 × 10^4^ cells/well) in a 96-well transparent plate and incubated at 37°C with 5% CO_2_ overnight. The next day, cells were subjected to serum starvation for 6 hours, followed by treatment with compound for 2 or 6 hours. Following incubation, cells were lysed and pAMPK detected using the chromogenic antibody binding pairs according to the manufacturer’s directions.

### Animals.

Sprague-Dawley rats, male, weighing between 100 and 350 g, were purchased from Charles River Labs. Only male rats were studied since NDI due to V2R mutations is an X-linked disorder. Cre (+) and Cre (–) V2R-floxed mice (20), between 20 and 40 g, were originally obtained from Jurgen Wess (NIDDK, NIH, Bethesda, Maryland, USA) and now are bred and housed in the Emory Animal Care facility.

Treatment of NDI rats with NDI-5033 (Figure 2) involved the following: rats were fed with water and standard rat chow (Purina Lab Diet, 5001) ad libitum. After collection of a 24-hour urine sample for basal osmolality determination, rats received daily tolvaptan (Otsuka Pharmaceutical Co.) to inhibit the V2R at 10 mg/kg/day suspended in 1% HPMC (MilliporeSigma) in water to produce NDI (5). After confirmation of V2R blockade and development of NDI in 1–3 days, rats were separated into control rats, which continued to receive tolvaptan, and NDI-5033–treated rats, which received tolvaptan and various doses of the drug also given by gavage in the HPMC (NDI-5033[P1]) or received lipid suspension media (NDI-5033[P2]; see below) for 4 to 10 days. All animals were housed in metabolic cages during the experimental period to facilitate collection of 24-hour urine samples. At the end of the protocol, animals were sacrificed by decapitation.

### Oral delivery of NDI-5033(P1).

NDI-5033(P1) in powdered form was added to 1% HPMC in water and stirred at room temperature for 30 minutes prior to giving it to the rats by gavage. The concentration of the suspension was adjusted to provide enough drug to reach the dosing requirements in a volume that was acceptable for the rat body size. For example, for a 0.2 kg rat receiving a dose of 10 mg/kg, 24 mg of the drug would be suspended in 3 mL, and each rat would receive 250 μL.

### Oral delivery NDI-5033(P2).

The method of synthesis and purification of the drug substance was changed to facilitate scale up and to improve therapeutic levels. This led to a form with lowered solubility in water. NDI-5033(P2) was reformulated in a lipid medium containing 20% transcutol, 40% labrasol, and 40% labrafil (Gattefossé) in water and used in that vehicle thereafter. The lipid medium was made in 50 mL stocks stored at room temperature and vortexed thoroughly before each use. For example, for a 200g rat, the gavage volume was 200–400 μL. At 10 mg/kg body weight/day, each rat received 2 mg tolvaptan dissolved in 250 μL lipid. For example, for 10 rats, 32 mg tolvaptan was dissolved in 4 mL lipid, vortexed, and separated into 2 × 2 mL volumes. To one of these, the appropriate amount of NDI-5033 was added to achieve the mg/kg/250 μL dose in the dosing studies. The solution was placed in a glass vial with screw cap, placed on a heated stirrer, and heated to 70°C–80°C with stirring for 20 minutes or until all drug was dissolved into the lipid medium. The solution was allowed to cool to room temperature before administration. The NDI-5033(P2)/lipid formulations were made daily.

### NDI-5033 dose-response protocol.

A timeline of this protocol is given in Figure 2. The same protocol was followed for both NDI-5033(P1) and NDI-5033(P2), with the vehicle being the difference between the studies. Rats were placed in metabolic cages, and 24-hour urine samples were collected to determine basal urine osmolality. Rats were then given tolvaptan suspended in HPMC at a concentration that allows provision of 10 mg/kg in a volume of 300–500 μL of feeding solution by oral gavage. Urine osmolality was followed for 3–4 days to ensure establishment of NDI. Half of the rats were then designated as the experimental group and fed with tolvaptan + NDI-5033 (at doses indicated in Results) in HPMC, while the other half continued to receive tolvaptan alone. Feeding was performed at approximately the same time each day. At the end of the experiment, rats were sacrificed by decapitation.

### NDI-5033 in V2R-KO mice.

V2R–KO was induced in genetically modified mice by feeding tamoxifen (TD.130859, Envigo) as described before (5, 20). Mice were determined to have successful KO of V2R when their urine osmolality fell below 300 mOsmol/kg H_2_O. Mice were given NDI-5033(P1) suspended in HPMC by i.p. injection at doses detailed in Results. Hourly and 24-hour urine samples were collected to determine the effect on concentrating ability. Mice were sacrificed by cervical dislocation.

### Urine Analysis.

Twenty-four–hour urine samples were collected from the rats daily. Hourly and daily urine were collected from the mice. Urine osmolalities were measured using a Wescor 5520 Vapor Pressure Osmometer (Wescor).

### Exercise protocol.

Rats were weighed and housed in metabolic cages, and urine was collected for a period of 24 hours to determine osmolality (day 1). All rats were given tolvaptan to produce NDI (day 2). NDI was verified by measuring urine osmolality (days 3–4). NDI-5033(P1), suspended in HPMC, was given to half of the rats daily by gavage feeding beginning on day 4 at a dose of 10 mg/kg/day. Urine was collected each morning, and osmolality was determined. Exercise was initiated on day 12. Rats were fed normally overnight. The next morning, rats were given NDI-5033; the food was removed and withheld for 3 hours. Blood (about 10 μL) was collected by tail vein nick and tested for blood glucose levels using a One Touch Ultra 2 glucometer (LifeScan Inc.). Next, the rats were exercised for 30 minutes on a treadmill at a speed of 12 m/min with 1.5% inclination, consistent with other aerobic exercise treadmill studies in rats (24, 25). Following the exercise, blood glucose levels were again determined, after which, food was restored. The same protocol was followed for 4 successive days.

### Statistics.

All data are presented as mean ± SEM. A Student’s 2-tailed *t* test was used for comparisons of 2 groups. Unpaired analysis was used for most comparisons, but paired analysis was performed with V2R mouse experiments because they are before-and-after determinations in the same animal. One-way ANOVA, followed by a Tukey-Kramer multiple-comparisons test was used for comparisons of more than 2 groups. Statistical differences with *P <* 0.05 were considered significant.

### Study approval.

All animal surgical protocols and procedures were approved by the Emory IACUC (protocol number PROTO201800110) and adhere to NIH standards for animal use.

## Author contributions

JDK designed and conducted experiments, analyzed data, and wrote the manuscript. IK designed reagents, analyzed AMPK activator PK data, and cowrote the manuscript. RP designed and analyzed AMPK activation pharmacokinetic data. RAH provided reagents and reviewed data. LML performed experiments and collected data. ELR performed experiments, collected data, and organized data. JMS designed study and experiments, analyzed data, and wrote the manuscript.

## Figures and Tables

**Table 1:**
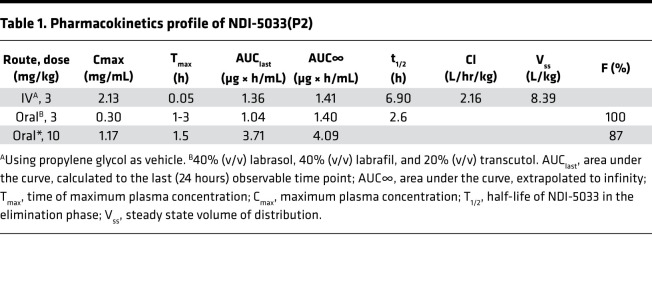
Pharmacokinetics profile of NDI-5033(P2)

**Table 2:**
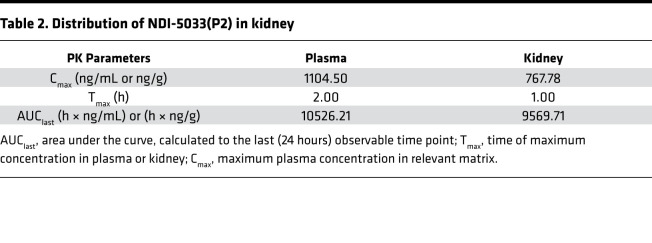
Distribution of NDI-5033(P2) in kidney

## Version 1 03/16/2021

In-Press Preview

## Version 2 04/22/2021

Electronic publication

## References

[1] Sands JM, et al. Urine Concentration and Dilution. In: Yu ASL, et al., eds. *Brenner and Rector’s The Kidney*. Elsevier; 2020:274-302.

[2] Sands JM, Klein JD (2016). Physiological insights into novel therapies for nephrogenic diabetes insipidus. Am J Physiol Renal Physiol.

[3] Sands JM, Bichet DG (2006). Nephrogenic diabetes insipidus. Ann Intern Med.

[4] Klein JD (2016). Metformin, an AMPK activator, stimulates the phosphorylation of aquaporin 2 and urea transporter A1 in inner medullary collecting ducts. Am J Physiol Renal Physiol.

[5] Efe O (2016). Metformin improves urine concentration in rodents with nephrogenic diabetes insipidus. JCI Insight.

[6] Rena G (2017). The mechanisms of action of metformin. Diabetologia.

[7] Khanna I, Pillarisetti S, inventors; Methods and compositions for treatment of diabetes and dyslipidemia. US Patent 8,623,897. January 7, 2014

[8] Watt MJ (2006). Fatty acids stimulate AMP-activated protein kinase and enhance fatty acid oxidation in L6 myotubes. J Physiol.

[9] Za’tara G (2008). AMPK activation by long chain fatty acyl analogs. Biochem Pharmacol.

[10] Bisgaier CL (1998). A novel compound that elevates high density lipoprotein and activates the peroxisome proliferator activated receptor. J Lipid Res.

[11] Woodworth-Hobbs ME. *The Effects of Omega-3 Polyunsaturated Fatty Acids on AMPK Activation and Lipid Metabolism in Skeletal Muscle.* Master’s thesis. West Virginia University; 2009.

[12] Verbalis JG. Disorders of Water Balance. In: Yu ASL, et al., eds. *Brenner and Rector’s The Kidney*. Elsevier; 2020:473.

[13] Bonnardeaux A, Bichet DG. Inherited Disorders of the Renal Tubule. In: Yu ASL, et al., eds. *Brenner and Rector’s The Kidney*. Elsevier; 2020:1485–1487.

[14] Bouley R (2005). Stimulation of AQP2 membrane insertion in renal epithelial cells in vitro and in vivo by the cGMP phosphodiesterase inhibitor sildenafil citrate (Viagra). Am J Physiol Renal Physiol.

[15] Sanches TR (2012). Sildenafil reduces polyuria in rats with lithium-induced NDI. Am J Physiol Renal Physiol.

[16] Cheung PW (2016). EGF receptor inhibition by erlotinib increases aquaporin 2-mediated renal water reabsorption. J Am Soc Nephrol.

[17] Li W (2011). Simvastatin enhances aquaporin-2 surface expression and urinary concentration in vasopressin-deficient Brattleboro rats through modulation of Rho GTPase. Am J Physiol Renal Physiol.

[18] Procino G (2016). Simvastatin increases AQP2 urinary excretion in hypercholesterolemic patients: A pleiotropic effect of interest for patients with impaired AQP2 trafficking. Clin Pharmacol Ther.

[19] Procino G (2014). Combination of secretin and fluvastatin ameliorates the polyuria associated with X-linked nephrogenic diabetes insipidus in mice. Kidney Int.

[20] Li JH (2009). A selective EP4 PGE2 receptor agonist alleviates disease in a new mouse model of X-linked nephrogenic diabetes insipidus. J Clin Invest.

[21] Zhang Y (2015). P2Y12 receptor localizes in the renal collecting duct and its blockade augments arginine vasopressin action and alleviates nephrogenic diabetes insipidus. J Am Soc Nephrol.

[22] de Groot T (2016). Acetazolamide attenuates lithium-induced nephrogenic diabetes insipidus. J Am Soc Nephrol.

[23] de Groot T (2017). Lithium-induced NDI: acetazolamide reduces polyuria but does not improve urine concentrating ability. Am J Physiol Renal Physiol.

[24] Pitcher MH (2018). The impact of exercise in rodent models of chronic pain. Curr Osteoporos Rep.

[25] Portier H (2020). Does physical exercise always improve bone quality in rats?. Life (Basel).

